# Bisphenol A enhances adipogenic signaling pathways in human mesenchymal stem cells

**DOI:** 10.1186/s41021-020-00150-6

**Published:** 2020-03-11

**Authors:** Amin Salehpour, Farzad Shidfar, Mehdi Hedayati, Asal Neshatbini Tehrani, Ali Asghar Farshad, Saeed Mohammadi

**Affiliations:** 1 Occupational Health Research Center, School of Public Health, Iran University of Medical Sciences, Tehran, Iran; 2 Department of Nutrition, School of Public Health, Iran University of Medical Sciences, Shahid Hemmat Highway, Tehran, Iran; 3 Cellular and Molecular Endocrine Research Center, Research Institute for Endocrine Sciences, Shahid Beheshti University of Medical Sciences, Tehran, Iran; 4 Department of Nutrition, School of Paramedicine, Ahvaz Jundishapur University of Medical Sciences, Ahvaz, Iran; 5 Department of Biostatistics, School of Public Health, Iran University of Medical Sciences, Tehran, Iran

**Keywords:** Bisphenol A, Adipogenesis, Human adipose-derived mesenchymal stem cells, Gene expression

## Abstract

**Background:**

The endocrine disruptor Bisphenol-A (BPA), has been involved in dysregulating adipose tissue development and increasing the risk of obesity. The objective of this experiment was to investigate whether treatment of human mesenchymal stem cells with BPA could modulate adipogenesis and adipocyte differentiation.

**Methods:**

In this experimental study, the human adipose-derived mesenchymal stem cells (hASCs) were cultured for 2 weeks with continuous exposure to 10^− 10^ M or 10^− 8^ M concentrations of BPA. The extent of triglyceride accumulation was visualized by Oil Red O staining. To evaluate BPA effect on the expression levels of key adipogenic trascripotion factors and proteins, we used Quantitative reverse transcriptase-polymerase chain reaction (qRT-PCR) and ELISA.

**Results:**

The results presented a dose-dependent triglyceride accumulation in treated cells with BPA. Additionally, we observed that BPA induced transcription of the Peroxisome proliferator-activated receptor-gamma (PPARγ), CCAAT-enhancer-binding protein-alpha (C/EBPα), CCAAT-enhancer-binding protein-beta (C/EBPβ), sterol regulatory element-binding protein-1c (SREBP1c), Fatty acid synthase (FASN), and lipoprotein lipase (LPL); BPA suppressed the expression of Fatty acid binding protein-4 (FABP4) and Estrogen receptor-beta (ERβ).

**Conclusions:**

Our findings supported the hypothesis that BPA enhances adipogenic differentiation thereby may play a role in development of obesity and dysregulation of metabolic homoeostasis.

## Introduction

In recent years, the prevalence of obesity has risen significantly to become one of the epidemic health problems and fostering growing comorbidities including insulin resistance, cardiovascular diseases, carcinogenesis, and infertility [1, 2].

While diet and a sedentary life style have obviously contributed to the increasing rate of obesity, there is mounting literature to propose that exposure to environmental chemicals, known as obesogens, may be cooperating to dramatic increase in prevalence of obesity [3–5]. Endocrine disruptors possibly act as obesogens, by stimulating adipogenesis via promoting fat accumulation or developing a positive energy balance [6, 7]. Endocrine disrupters can interfere with the endocrine system by mimicking or modifying the synthesis, transportation, metabolism, excretion or action of endogenous hormones [8, 9].

Bisphenol A (BPA) is a well-known estrogen-like activity chemical that everyone may expose to because it is used in manufacturing of a wide range of products such as food packaging, feeding bottles, the epoxy based lining of canned foods, and more [10–12]. BPA can be released from these materials into food and water and then into human body [13, 14]. Nanomolar levels of BPA can be detected in human serum, amniotic fluid, milk, semen and urine. Mechanistically, the action of BPA is related to its homology with estrogen. BPA prompts the activation of estrogen receptors α (ER-α) and β (ER-β) [15, 16].

Several studies showed that treatment of 3T3-L1 fibroblasts with BPA results in increasing the adipocyte differentiation and fat accumulation by stimulating the activity of lipoprotein lipase (LPL), Glyceraldehyde-3-phosphate dehydrogenase (GPDH), and peroxisome proliferator- activated receptor-γ (PPARγ), which are all engaged in lipid metabolism and storage [17–21]. Interestingly, 3T3-F442A cells treated with BPA showed boosted basal and insulin-stimulated glucose uptake via increasing the gene expression of Glucose transporter type 4 (GLUT4). It is also confirmed that BPA has an important role in final differentiation of preadipocytes into adipocytes by increasing the mRNA level of LPL and Adipocyte protein 2 (aP2) [21–23].Thus, the present study was conducted to evaluate the effects of BPA on adipogenesis in human adipose-derived mesenchymal stem cells.

## Materials and Methods

### Cell culture and differentiation

Human adipose-derived mesenchymal stem cells (hASCs) were obtained from human cells bank of Iranian Biological Resource Center laboratory (Tehran, Iran). In this experimental study, hASCs were cultured in Dulbecco’s modified Eagle’s medium (DMEM) supplemented with 10% FBS, 2% Glutamine, 100 IU/ml Penicillin and 100 IU/ml Streptomycin. Cultures were maintained in humidified atmosphere of 95% air and 5% CO_2_ at 37 °C. The media was changed with fresh growth media every 48 h. After 2 days post confluence, differentiation was initiated. For adipocyte differentiation, cells in early passage (not exceeding 4 passage) were seeded at 5.04231 cells/ml, a density pre-optimized for adipogenic differentiation. After 24 h, confluent cultures (Day 0) were stimulated to differentiate with adipocyte differentiation medium (Gibco,UK) containing concentrations of 0.5 mM 3-isobutyl-3-methylxanthine (IBMX), 1 mM Dexamethasone (DEX), and 5 mg/ml human insulin. At the time of induction of differentiation, mesenchymal pre-adipocytes were treated with 10^− 10^ or 10^− 8^ M of BPA for 2 weeks. Wells were divided into three experimental groups with at least three parallel wells in each group: (i) 10^− 10^ M of BPA with induction; (ii) 10^− 8^ M of BPA with induction; (iii) control with induction. After 7 days, media was changed to an adipocyte maintenance medium (Gibco,UK) and cultured for a further 7 days. All these mix changes were done in parallel in control cells. Cells were collected and analyzed at hours 1, 3, and days 1, 3, 6, and 14 during differentiation. Unless otherwise stated, all chemicals were from Sigma-Aldrich.

### Quantitative Real-Time Polymerase Chain Reaction (qRT-PCR)

Total RNA was extracted using the TRIzol reagent (Sigma-Aldrich) and then, according to manufacturer’s instructions, RNA was reverse transcribed to cDNA using the Superscript II Reverse Transcriptase kit (Invitrogen). qRT-PCR was performed using the Step One Plus Real-Time PCR System (Applied Biosystems) and SYBR Premix Ex Taq II, Tli RNaseH Plus (Takara, Japan). Primer pairs for PPARγ, C/EBPα, C/EBPβ, SREBP1c, FASN, LPL and Insulin induced gene-2 (INSIG2) were designed using the Primer-blast software (NCBI, USA). The 2^-ΔΔCt^ method was used to calculate fold changes of mRNA expression levels. The genes were normalized to those of GAPDH. The name and sequence of the primers, the sizes, and annealing temperatures for each pair are listed in Table 1.

**Table 1:** The name and sequence of the primers, the sizes, and annealing temperatures for each pair

Gene	Size (bp)	Sequence (5′ → 3′)	Annealing temperature (°C)
GAPDH	113	F:CATGAGAAGTATGACAACAGCCT R:AGTCCTTCCACGATACCAAAGT	58
PPARγ	80	F:CAGAAATGCCTTGCAGTGGG R:AACAGCTTCTCCTTCTCGGC	59
CEBPα	94	F:TATAGGCTGGGCTTCCCCTT R:AGCTTTCTGGTGTGACTCGG	60
CEBPβ	154	F:TTTGTCCAAACCAACCGCAC R:GCATCAACTTCGAAACCGGC	59
SREBP1c	117	F:TCTCAGTCCCCTGGTCTCTG R:ATAGGCAGCTTCTCCGCATC	59
INSIG2	114	F:AGTGGTCCAGTGTAATGCGG R:TGGATAGTGCAGCCAGTGTG	60
LPL	137	F:GCTCAGGAGCATTACCCAGTGTC R:GCTCCAAGGCTGTATCCCAAGA	63
FASN	107	F:ATTCTGCCATAAGCCCTGTC R:CTGTGTACTCCTTCCCTTCTTG	57

*GAPDH* Glyceraldehyde-3-phosphate dehydrogenase, *PPARγ* Peroxisome proliferator-activated receptor-gamma, *C/EBPα* CCAAT-enhancer-binding protein-alpha, *C/EBPβ* CCAAT-enhancer-binding protein- beta, *SREBP1c* Sterol regulatory element-binding protein-1c, *INSIG2* Insulin induced gene-2, *FASN* Fatty acid synthase, *LPL* lipoprotein lipase

### Oil Red O staining

To assess cellular triglyceride accumulation, adipocyte cell monolayers were gently rinsed three times with iced Phosphate-Buffered Saline (PBS) and fixed with 4% Paraformaldehyde for 30 min. After fixation, the cells were washed three times and stained with Oil Red O solution (ORO) for 15 min at room temperature. Cells were washed again three times with PBS to remove unbound staining. ORO-stained adipocytes were observed under a microscope (Olympus, Tokyo, Japan) and digital images were captured at 100X magnification.

### Protein assay

Cells were washed in PBS and lysed in buffer containing 50 mM Tris, 150 mM sodium chloride (NaCl), 1% IGEPAL, 5 mM EDTA (all from Sigma-Aldrich), and protease inhibitor cocktail (Roche Diagnostics, Laval, QC, Canada). Tissue Fatty acid binding protein-4 (FABP4), GLUT4, and ERβ protein concentrations were determined at days 7 and 14, using the related research specific enzyme-linked immunosorbent assay (ELISA) kits. Tissue FABP4 concentration was determined using Human FABP4 ELISA kit, coefficient of variability (CV) was calculated the intra assay precision (5.5%). GLUT4 concentration was determined by Human GLUT4 ELISA kit, CV% was calculated the intra assay precision (5.8%). ERβ concentrations was determined using Human ERβ ELISA kit, CV% was calculated the intra assay precision (6.1%). All the research kits were prepared from ZellBio GmbH, Ulm, Germany and the microplate reader was Epoch Model, BioTek, Vermont, USA.

### Statistical analysis

Data are expressed as means±standard deviation (SD). The mRNA expressions were determined by analysis of variance (ANOVA) with repeated measures and 2-tailed Student t tests (SPSS 25 for Windows, standard version; SPSS Inc., Chicago, IL, USA).using GraphPad (GraphPad Software) and the protein expressions were determined by Kruskal–Wallis one-way analysis of variance and Dunn’s multiple comparison test. Means were considered statistically different when *p* values were less than 0.05.

## Results

**Fig. 1:**
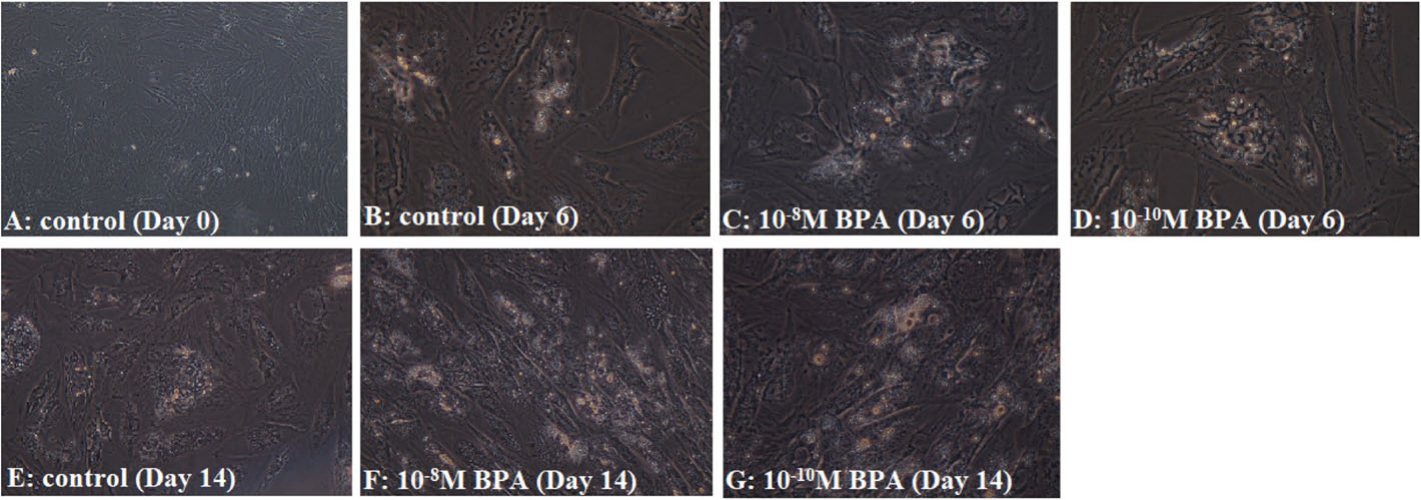
Oil Red O staining of human adipose-derived mesenchymal stem cells. Phase contrast image of adipocytes were taken by microscope (Olympus, Tokyo, Japan) and digital images were captured at 100X magnification. Following 14 days of treatment with BPA showed a significant increase in relative lipid vacuole staining compared with control group

**Fig. 2:**
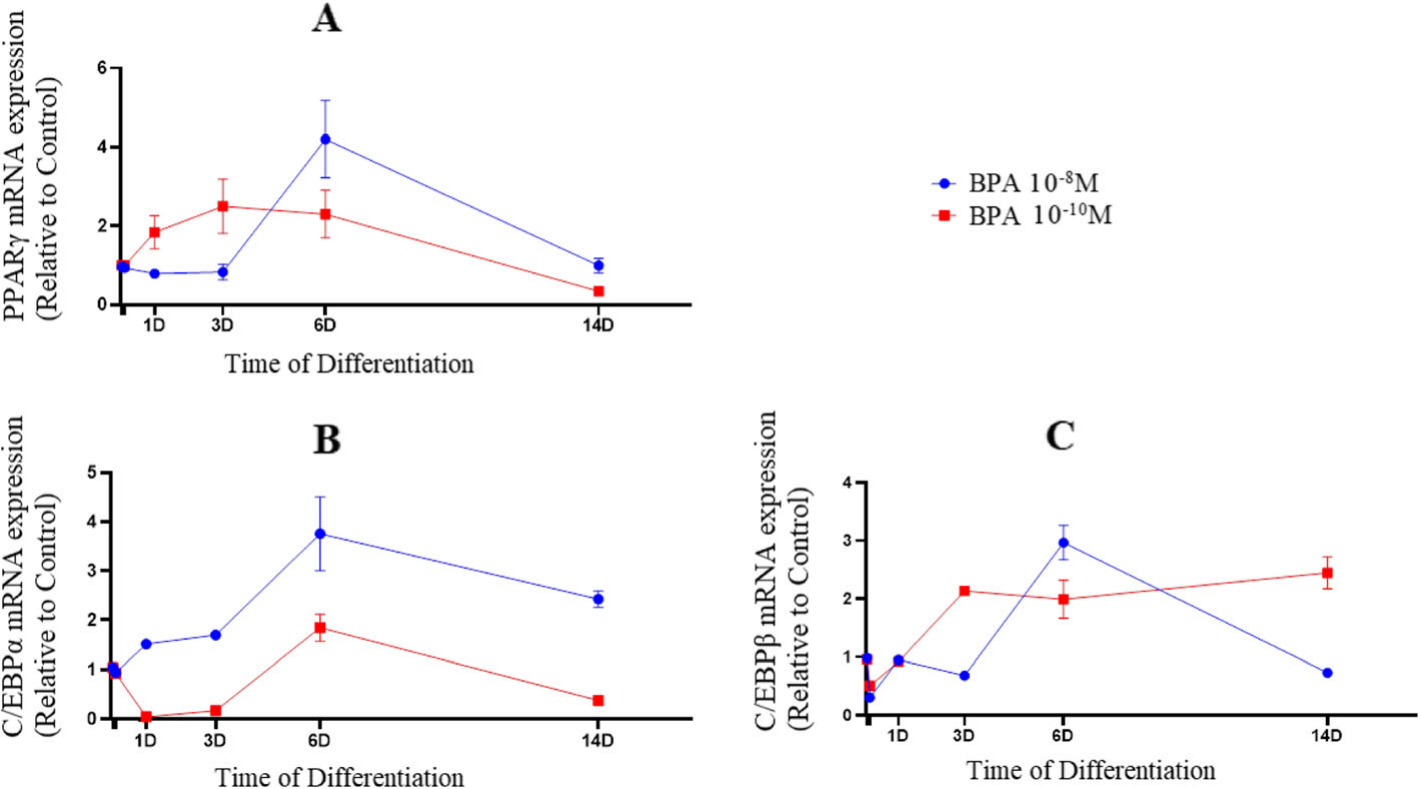
mRNA expression of PPARγ (**a**), C/EBPα (**b**) and C/EBPβ (**c**) in BPA groups during adipogenic differentiation.The relative qPCR values were corrected to GAPDH expression levels and normalized with respect to controls on each time

To visualize adipogenic differentiation and lipid accumulation, the morphology of the hASC was displayed at days 6 and 14 of differentiation (Fig. 1). To distinguish the adipogenesis augmentation by BPA, the mRNA expression of main adipogenic enzymes and transcription factors were calculated using qPCR. Mitotic clonal expansion phase (days 0–2) and terminal differentiation (days 4–6) are described as the initial and late stages of adipocyte differentiation respectively [24, 25]. BPA (10^− 10^ M) inclined to upregulate mRNA expression of PPARγ (*F* = 13.03; *P* = 0.042) in initial period of the experiment. Similarly, mRNA level of PPARγ was increased (*F* = 30.9; *P* = 0.026), but did not in the late stage of differentiation in response to 10^− 8^ M BPA (Fig. 2a).

The time dependent effects on mRNA levels of main transcription factors during differentiation were evaluated to assess the early or late adipogenic signaling effects of BPA. At 6 days post treatment, the mRNA expression of C/EBPα was statistically significant increased in response to BPA concentrations, but continued exposure of the cells to 10^− 10^ M (*F* = 112.2; *P* = 0.008) or 10^− 8^ M BPA (*F* = 33.5; *P* = 0.024) caused a significant decline in the mRNA levels of C/EBPα at 14 days post treatment (Fig. 2b). Interestingly, while the mRNA expression of C/EBPβ, known as an early adipogenic transcription factor, was decreased in late phase of adipogenesis in 10^− 8^ M BPA (*F* = 172.5; *P* = 0.006), higher expression was maintained at day 14 in the treated cells with 10^− 10^ M BPA (*F* = 82.2; *P* = 0.012), (Fig. 2c).

**Fig. 3:**
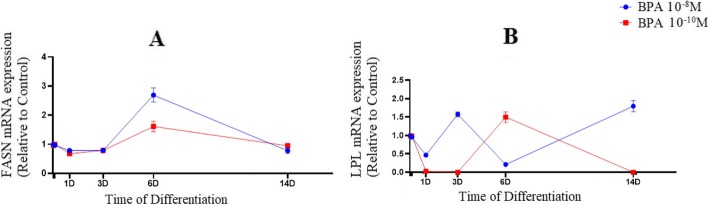
mRNA expression of FASN (**a**) and LPL (**b**) in BPA groups during adipogenic differentiation.The relative qPCR values were corrected to GAPDH expression levels and normalized with respect to controls on each time

Markedly, mRNA levels of FASN, known as a marker of de novo lipogenesis, were increased significantly at day 6 from the initiation of adipogenic differentiation in treated cells with 10^− 8^ M and 10^− 10^ M BPA (*F* = 143.8; *P* = 0.003) and (*F* = 57.5; *P* = 0.012) respectively, while a significant decline was perceived after day 6 (Fig. 3a). Intriguingly, BPA (10^− 8^ M) exposure resulted in dramatic fluctuations in LPL expression (*F* = 248.8; *P* < 0.001), known as a late marker of adipogenesis, during differentiation. While, mRNA expression of LPL was decreased in response to 10^− 10^ M BPA after 6 days post treatment (*F* = 366.9; *P* = 0.003). In 10^− 8^ M BPA treatment, mRNA level of LPL was increased during the later period of differentiation (day 6+) (Fig. 3b).

**Fig. 4:**
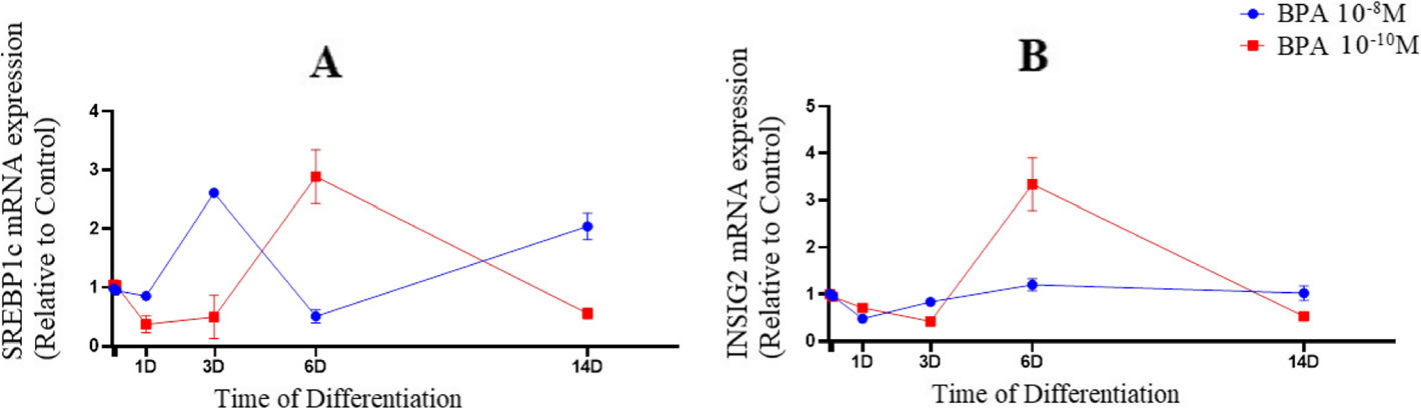
mRNA expression of SREBP1c (**a**) and INSIG2 (**b**) in BPA groups during adipogenic differentiation.The relative qPCR values were corrected to GAPDH expression levels and normalized with respect to controls on each time

Following BPA (10^− 10^ M) exposure, mRNA expression of SREBP1c was significantly increased (*F* = 50.8; *P* = 0.002) in 6 days post treatment (Fig. 4a). Additionally, mRNA levels of SREBP1c displayed a significant decrease (*F* = 161.05; *P* = 0.006) upon BPA (10^− 8^ M) exposure on day 6 and followed by an increase in the expression of SREBP1c (Fig. 4a).

On day 6, treatment with 10^− 10^ M BPA significantly upregulated mRNA levels of INSIG2 (*F* = 69.03; *P* = 0.014), known as an intermediate regulator between PPARγ and SREBP1c (Fig. 4b). Conversely, the expression levels of INSIG2 reached to the plateau (*F* = 19.3; *P* = 0.039) by 10^− 8^ M of BPA (Fig. 4b).

To evaluated the effect of BPA exposure on the levels of FABP4, other late marker of adipogenesis, we tested the ability of 10^− 10^ M or 10^− 8^ M concentrations of BPA to induce the adipogenic differentiation of hASCs at day 6 and 14. The results presented a statistically significant decline in FABP4 protein levels relative to control group (Table 2). Furthermore, the protein levels of GLUT4 were decreased upon BPA exposure compared to the control (Table 2).

**Table 2:** Comparison of protein expression in BPA groups vs. control

Proteins	Time	Group	Mean^a^	Standard Deviation	Result
FABP4	Day 6	Control	0.27	0.03	X = 6.5 df = 2 *P*-value = 0.03*
		10^−8^ M BPA	0.23	0.05	
		10^−10^ M BPA	0.18	0.02	
	Day 14	Control	0.32	0.02	X = 9.04 df = 2 *P*-value = 0.01*
		10^−8^ M BPA	0.18	0.006	
		10^−10^ M BPA	0.2	0.01	
GLUT4	Day 6	Control	0.17	0.03	X = 6.5 df = 2 *P*-value = 0.03*
		10^−8^ M BPA	0.14	0.03	
		10^−10^ M BPA	0.11	0.01	
	Day 14	Control	0.20	0.01	X = 9.04 df = 2 *P*-value = 0.01*
		10^−8^ M BPA	0.11	0.003	
		10^−10^ M BPA	0.12	0.009	
ERβ	Day 6	Control	1.1	0.73	X = 3.84 df = 2 *P*-value = 0.1^†^
		10^−8^ M BPA	0.92	0.22	
		10^−10^ M BPA	0.73	0.09	
	Day 14	Control	1.29	0.09	X = 9.4 df = 2 *P*-value = 0.009*
		10^−8^ M BPA	0.74	0.02	
		10^−10^ M BPA	0.80	0.06	

*FABP4* Fatty acid binding proteins-4, *GLUT4* Glucose transporter-4, *ERβ* Estrogen receptor beta

^a^ng/mg total protein

*Mean values were significantly different between the groups (*P* < 0.05)

^†^ Mean values were not significantly different between the groups (*P* > 0.05)

To report whether ERβ is involved in the action of BPA through differentiation of human mesenchymal stem cells, we measured protein levels of ERβ. While in the treated cells with BPA the expression of ERβ inhibited, the protein expression of ERβ was increased in control group after 2 weeks of differentiation (Table 2).

## Discussion

BPA is one of the most consumed synthetic compounds that is leading to proximate abundant exposure among the population globally and the effect on human metabolic health represented by the environmental level of BPA have been considerably investigated [6]. Mounting literature support the environmental obesogen hypothesis that proposes involvement of BPA in adipose tissue development and function [26, 27]. Evidence demonstrates that BPA could enhance the adipogenesis [21, 22]. Whereas the most epidemiological studies demonstrate a significant relationship between BPA and obesity [28, 29], in vivo and in vitro studies have provided controversial findings [30, 31].

The objective of current study was to examine the consequences of low doses of BPA on adipogenesis in the human adipose-derived mesenchymal stem cells as the in vitro system. Our findings showed that BPA markedly upregulated the gene expression of C/EBPα, C/EBPβ, PPARγ, LPL, FASN, and SREBP1c. Because of the amplified expression of this adipogenic transcription factors and enzymes, there may be an increase in the adipogenesis and lipid accumulation in response to BPA. We observed that BPA treatment declined the expression of ERβ, GLUT4, and FABP4.

A number of signaling pathways have been recognized to describe the obesogenic properties of BPA [32–34]. It has been testified that BPA interacts with Nuclear Receptors (NR) such as Retinoid X Receptor (RXR), Peroxisome Proliferator-Activated Receptors (PPAR), Estrogen Receptors (ER), Thyroid Receptors (TR), and Glucocorticoid Receptors (GR) [35, 36]. Thus, adipogenic differentiation and lipid accumulation are induced by BPA through stimulation of these receptors [37, 38]. Data have pointed out that BPA acts as an obesogene by triggering signaling over the ER [39–41]. Ohlstein et al [42] indicated that BPA induced the expression of ER and the other adipogenic factors PPARγ, C/EBPα, LPL, and insulin-like growth factor-1 (IFG1) throughout human stem cells differentiation. Boucher et al [43] showed that the ER agonist estradiol did not affect the differentiation of human preadipocytes, consistent with previous researches showing that estrogen had no stimulatory effect on adipogenesis [44]. BPA is recognized to have estrogenic characteristics and bind to the estrogen receptors. However, BPA has presented contradictory consequences of estradiol on differentiation. ER antagonist (2R,3R)-rel-3-isopropylamino-1-(7-methylindan-4-yloxy)-butan-2-ol hydrochloride **(**ICI) was able to suppress BPA-induced adipogenesis by downregulating expression of FABP4 protein levels. Thus, it has been proposed that BPA may possibly regulate adipogenesis via a non-classical ER pathway [45, 46]. In the present study, we showed that hASCs exposure to BPA resulted in increased mRNA expression of PPARγ, C/EBPα, and C/EBPβ. It is postulated that BPA interacts with ER and translocates to nucleus where the mRNA expression of main adipogenic markers including PPARγ, C/EBPα, and LPL is augmented and facilitated the differentiation of stem cells to mature adipocytes [42]. In the existence of GR, BPA may possibly enhance the transcriptional activity of C/EBP family members. It has been formerly presented that GR boosted the transcriptional activity of C/EBPβ on the C/EBPα promoter that was facilitated by DEX [47, 48].

Atlas et al [49] demonstrated that BPA promotes the adipogenic potential of the 3 T3-L1 cells at concentrations within the range reported in humans [50]. In addition, studies have reported the effects of BPA at low concentrations on reducing adipokines production in adipose tissue [30, 51]. It is well established that the adipogenesis process is driven by a cascade of adipogenic transcriptional markers including master adipogenic regulators such as PPARγ and C/EBPs. Consistent with our findings, further studies have reported that BPA treatment enhanced expression of PPARγ and LPL in 3 T3-L1 cells [19, 21]. Animal model studies showed that prenatal exposure to BPA resulted in inducing PPARγ, C/EBPα, LPL and developing adipose tissue [31]. Consecutively, these transcriptional factors result in inducing the expression of proteins such a FABP4 that may play a role in insulin sensitivity, as well as glucose and lipid metabolism. Atlas et al [47] showed that BPA increased FABP4 protein levels dose dependently.

While additional studies indicated that BPA exposure promoted expression of LPL and PPARγ [19, 21]. Contrary to our results, Chamorro-Garcia et al [30] reported no effect in human BMSCs upon BPA treatment. In addition, Linehan et al [45] have found that BPA suppressed triglyceride accumulation by inhibiting mRNA expression of LPL. Interestingly, BPA had no effect on the expression level of PPARγ, C/EBPα, and FABP4 despite the upregulation of LPL gene, suggesting that LPL may possibly promote the triglyceride accumulation independently [45]. BPA had no effect on the mRNA expression of FAS, a key regulator of de novo lipogenesis, proposing a minimal role of de novo lipogenesis in hASCs [45].

## Conclusions

The present research has evidenced that when human mesenchymal stem cells were chronically treated with BPA, alterations in induction of adipogenic related genes happened. Further studies are needed for better understanding of the BPA molecular mechanisms in modifying adipogenesis and fat accumulation.

## Data Availability

Not applicable.

## Abbreviations

aP2: Adipocyte protein 2
BPA: Bisphenol-A
C/EBPα: CCAAT-enhancer-binding protein-alpha
C/EBPβ: CCAAT-enhancer-binding protein- beta
CV: Coefficient of variability
DEX: Dexamethasone
DMEM: Dulbecco’s modified eagle’s medium
ERβ: Estrogen receptor-beta
FABP4: Fatty acid binding protein-4
FASN: Fatty acid synthase
GAPDH: Glyceraldehyde-3-phosphate dehydrogenase
GLUT4: Glucose transporter type
GR: Glucocorticoid Receptors
hASCs: Human mesenchymal stem cells
IBMX: 3-isobutyl-3-methylxanthine
ICI: (2R,3R)-rel-3-isopropylamino-1-(7-methylindan-4-yloxy)-butan-2-ol hydrochloride
INSIG2: Insulin induced gene-2
LPL: Lipoprotein lipase
NR: Nuclear Receptors
PPARγ: Peroxisome proliferator-activated receptor-gamma
qRT-PCR: Quantitative real-time reverse transcription-PCR
RXR: Retinoid X Receptor
SREBP1c: Sterol regulatory element-binding protein-1c
TR: Thyroid Receptors
